# Ablation dynamics during laser interstitial thermal therapy for mesiotemporal epilepsy

**DOI:** 10.1371/journal.pone.0199190

**Published:** 2018-07-06

**Authors:** Walter J. Jermakowicz, Iahn Cajigas, Lia Dan, Santiago Guerra, Samir Sur, Pierre-Francois D’Haese, Andres M. Kanner, Jonathan R. Jagid

**Affiliations:** 1 Department of Neurological Surgery, University of Miami Miller School of Medicine, Miami, Florida, United States of America; 2 Department of Electrical Engineering and Computer Science, Vanderbilt University, Nashville, Tennessee, United States of America; 3 Epilepsy Division, Department of Neurology, University of Miami Miller School of Medicine, Miami, Florida, United States of America; George Washington University, UNITED STATES

## Abstract

**Introduction:**

The recent emergence of laser interstitial thermal therapy (LITT) as a frontline surgical tool in the management of brain tumors and epilepsy is a result of advances in MRI thermal imaging. A limitation to further improving LITT is the diversity of brain tissue thermoablative properties, which hinders our ability to predict LITT treatment-related effects. Utilizing the mesiotemporal lobe as a consistent anatomic model system, the goal of this study was to use intraoperative thermal damage estimate (TDE) maps to study short- and long-term effects of LITT and to identify preoperative variables that could be helpful in predicting tissue responses to thermal energy.

**Methods:**

For 30 patients with mesiotemporal epilepsy treated with LITT at a single institution, intraoperative TDE maps and pre-, intra- and post-operative MRIs were co-registered in a common reference space using a deformable atlas. The spatial overlap of TDE maps with manually-traced immediate (post-ablation) and delayed (6-month) ablation zones was measured using the dice similarity coefficient (DSC). Then, motivated by simple heat-transfer models, ablation dynamics were quantified at amygdala and hippocampal head from TDE pixel time series fit by first order linear dynamics, permitting analysis of the thermal time constant (*τ*). The relationships of these measures to 16 independent variables derived from patient demographics, mesiotemporal anatomy, preoperative imaging characteristics and the surgical procedure were examined.

**Results:**

TDE maps closely overlapped immediate ablation borders but were significantly larger than the ablation cavities seen on delayed imaging, particularly at the amygdala and hippocampal head. The TDEs more accurately predicted delayed LITT effects in patients with smaller perihippocampal CSF spaces. Analyses of ablation dynamics from intraoperative TDE videos showed variable patterns of lesion progression after laser activation. Ablations tended to be slower for targets with increased preoperative T2 MRI signal and in close proximity to large, surrounding CSF spaces. In addition, greater laser energy was required to ablate mesial versus lateral mesiotemporal structures, an effect associated with laser trajectory and target contrast-enhanced T1 MRI signal.

**Conclusions:**

Patient-specific variations in mesiotemporal anatomy and pathology may influence the thermal coagulation of these tissues. We speculate that by incorporating demographic and imaging data into predictive models we may eventually enhance the accuracy and precision with which LITT is delivered, improving outcomes and accelerating adoption of this novel tool.

## Introduction

Laser interstitial thermal therapy (LITT) is quickly changing our management of patients with brain tumors and epilepsy by offering a viable minimally-invasive surgical option. LITT’s recent resurgence is a result of advances in MRI thermal imaging that have improved real-time intraoperative monitoring of the lesion [1–4]. This is important because under-ablation may lead to treatment failure, while over-ablation can cause cognitive or neurologic deficits [1, 3–6]. Compared to open surgery, LITT patients experience less morbidity and have shorter hospital stays. However, as with any novel technique important gaps in our knowledge still exist. In the case of mesial temporal lobe epilepsy (mTLE), the most common epilepsy syndrome, recent series suggest lower seizure freedom rates for LITT (53–57%) vs. open surgery (60–80%) [1–4]. Improving seizure outcomes will not only require better understanding of laser ablation zones and their relation to epileptogenic areas, but also an improved ability to deliver LITT treatments with a millimetric level of precision.

A limitation to improving LITT is that not all brain areas respond to thermal stress in a similar manner, which impedes our ability to predict LITT treatment-related effects [7–12]. For example, given tissue inhomogeneities, heat may spread asymmetrically leading to inadequate ablation or increased threat to nearby functional tissues [11, 13, 14]. This may result in treatment failure or require return from the MRI suite to the operating room for laser repositioning [1, 15, 16]. Variability in tissue thermoablative properties is supported by animal and simulation studies, which suggest that tissues ablate differently depending on pathology and influences of local anatomy, such as the presence of nearby blood vessels or CSF spaces [10–12, 17–19].

Decisions during LITT are guided by Thermal Damage Estimate (TDE) maps, which are predictions of irreversible tissue damage calculated from gradient-recalled echo-derived thermal images using the Arrhenius rate process model, an estimate of permanent damage using each pixel’s temperature history [9, 12]. However, the accuracy of TDE maps at predicting LITT ablation zone boundaries is not clear. Although recent reports on the topic do suggest these estimates generally are accurate, variability has been observed [9, 20–22]. Limitations of these prior studies is their focus on brain tumors, where ablation targets vary tremendously in size, shape, and location, and lack of proper image normalization tools. Without adequate co-registration of LITT imaging data in a common reference it is difficult to assess the accuracy of TDE maps or to glean insights into patient- and procedure-specific variables that influence tissue ablation [23, 24].

The repeated use of the same ablation target for mTLE creates uniformity in tissue qualities and presents a unique model to study thermocoagulation in the human brain. Therefore, our goal was to investigate patient-specific differences in mesiotemporal laser ablative properties and identify correlates with patient demographics, preoperative imaging characteristics, or local anatomy that could eventually be helpful in predicting LITT ablations. First, using novel image normalization tools we examined the relationship of intraoperative TDE maps to ablation zone boundaries measured from immediate and 6-month delayed postoperative imaging [4, 23]. These data were then co-registered across patients to search for regional variations in TDE map predictive accuracy. Next, to gain a better understanding of variables that influence formation of these ablation zones we evaluated LITT dynamics by analyzing intraoperative Arrhenius-derived TDE videos on a pixel-by-pixel basis and modeling with best-fit exponential curves. Our data show diverse tissue responses to thermal energy, which appear to be correlated with certain variables measured from preoperative imaging and the surgical procedure.

## Materials and methods

### Patient selection

All University of Miami patients who underwent LITT for treatment of mTLE with a minimum of 6-months post-surgical follow-up were included, for a total of 30 subjects. Standard pre-surgical work-up included EEG monitoring, 3-Tesla thin-cut MRI scans using epilepsy protocol, interictal positron emission tomography, neuropsychological evaluation, and either functional MRI or Wada testing to lateralize language dominance. A subset of patients also required magnetoencephalography (MEG) as well as invasive monitoring. Following surgery, patients underwent repeat thin-cut MRI at 6-months. Institutional Review Board (IRB) approval and patient consent were obtained for all aspects of this study.

### Surgical procedure

All procedures were described in detail in prior publications [4, 6]. Briefly, a CRW (Plainsboro, NJ) stereotactic frame was placed and a thin-cut CT obtained and merged with the preoperative MRI to obtain frame-based target coordinates. The Medtronic (Minneapolis, MN) Visualase laser fiber was then stereotactically-guided using an occipital trajectory into the amygdalohippocampal complex (AHC). The patient was subsequently transported to the MRI suite for thermal ablation. A test dose at 30% maximal power always preceded the ablative doses (65–85% maximal power) in order to confirm proper laser placement. Typically, 3–5 serial ablations were used for each procedure.

### Image analyses

Videos of intraoperative TDE maps and ablation records were obtained postoperatively from the Visualase workstation. For the first 12 patients in our series only axial TDE maps were used during the procedure. For the remainder both axial and sagittal maps were used. The TDE map files of two patients were corrupted and could not be used. Therefore, we had axial TDE maps for 28 patients and sagittal TDE maps for 16 patients. Quantitative image analyses were performed using the Matlab (Natick, MA) Image Processing Toolbox.

**Fig 1:**
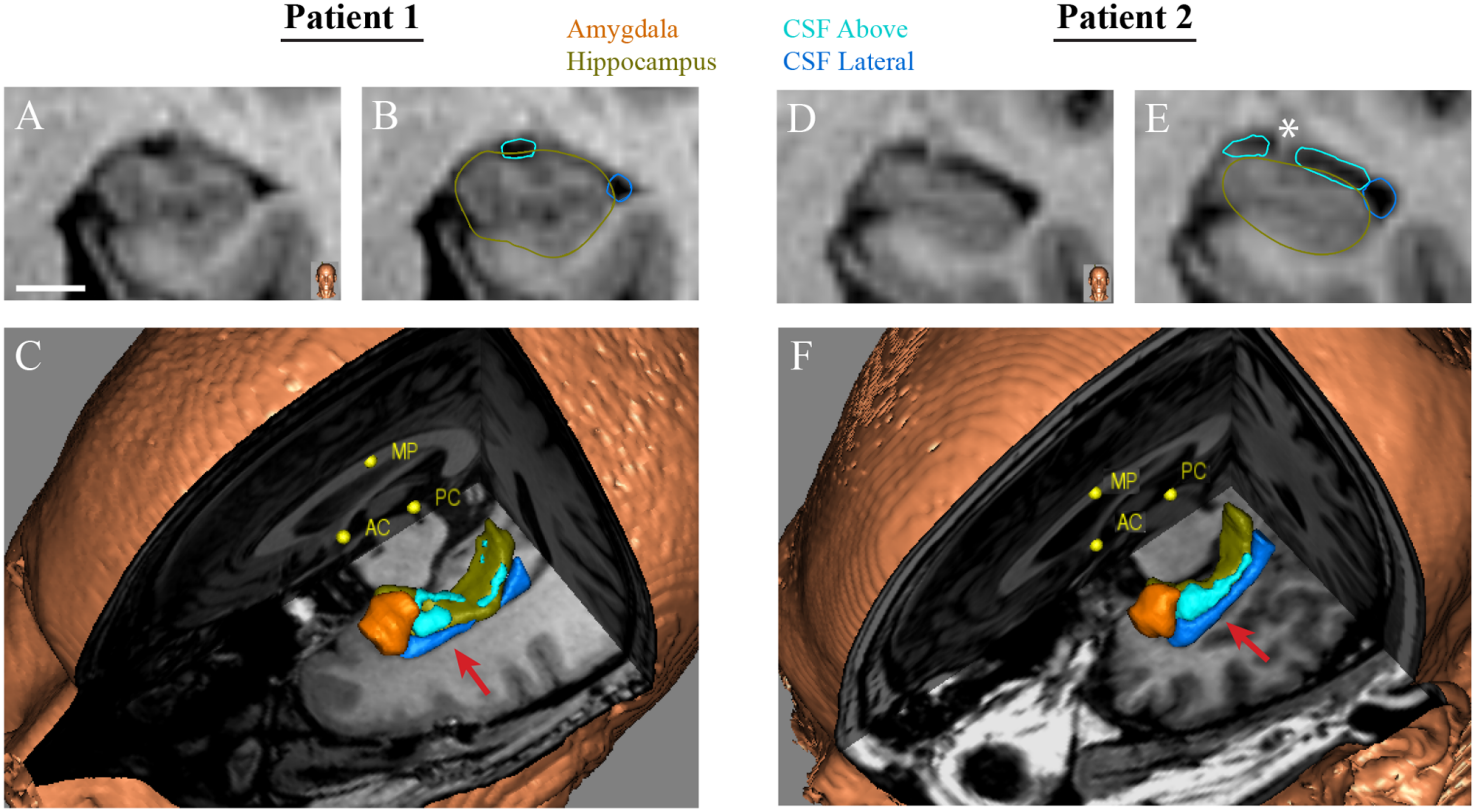
Preoperative volumetric analyses. (A) T1 MP-RAGE coronal cut through the hippocampal body for a representative patient. (B) Same image showing manually-traced borders of CSF_Above_ (light blue) and CSF_Lateral_ (dark blue) and automatically segmented hippocampal borders (green). (C) 3D model for the same patient showing relationship of CSF structures to amygdala (orange) and hippocampus (green). The red arrow shows the imaging cut used for A & B. (D-F) Same conventions as in A—C for a patient with larger CSF spaces. The white asterisks in E shows the vertical digitation of the hippocampus at its posterior end, which was used as a consistent anatomic landmark for laser position calculations. Scale bar– 5 mm. AC, anterior commissure; MP, midline point; PC, posterior commissure.

All volumetric data from preoperative imaging were acquired from non-deformed T1 MP-RAGE sequences using CranialSuite clinical software (Nashville, TN) [4, 6]. Immediate and 6-month delayed ablation volumes were manually traced from serial 1 mm coronal cuts by an investigator (LD) blinded to outcomes. Manual contrast thresholding was used as an adjunct to define ablation boundaries. Hippocampal and amygdala volumes were acquired automatically by deforming to each image an anatomic atlas derived from 7-Tesla (T) MRI data. If necessary, boundaries were then adjusted by an experienced investigator to be in accordance with accepted standards for hippocampal segmentation [25, 26]. CSF spaces surrounding the hippocampus were manually traced in 1 mm cuts from coronal images into “CSF above” and “CSF Lateral” compartments (Fig 1). “CSF Above” represents the volume of CSF immediately above the hippocampus, corresponding to the choroidal fissure and upper portion of the temporal horn of the lateral ventricle. “CSF Lateral” is the volume of CSF in the remainder of the temporal horn, lateral to the hippocampus. Laser position was measured from a coronal cut at the border of hippocampal head and body, always using the most posterior cut containing the vertical digitation of the hippocampus (Fig 1E). Superior—Inferior (S-I) position shows laser location relative to the cranial (value of 0) and caudal (value of 1) extents of the mesiotemporal lobe (including hippocampus, white matter and perirhinal cortex). Mesial—Lateral (M-L) position indicates location relative to the mesial (value of 0) and lateral (value of 1) walls of the hippocampus at the same coronal cut. “Axial angle” was measured from axial images as angle of the laser relative to the antero-posterior midline. “Sagittal angle” was measured from sagittal images as laser angle relative to a line dissecting the anterior and posterior commissures. With greater sagittal angle the laser points lower in the sagittal plane. Pixel intensities were computed for the hippocampus through a single coronal cut at the largest cross-section through the hippocampal head using our hospital-based Citrix (Fort Lauderdale, FL) clinical imaging software. All relevant MRIs and TDE videos are openly available on the CranialCloud [4, 6].

To compare TDE maps with ablation zone boundaries, pre-, intra- and post-operative MRIs were co-registered using CranialSuite, with the preoperative MRI used as the reference. The preoperative and 6-month postoperative MRIs were contrasted T1 MP-RAGE images with 1 mm slice thickness. The post-ablation intraoperative contrasted T1 MRIs either had similar 1 mm resolution or had 3 mm resolution with 1 mm thin cuts. To compare TDE maps with postoperative ablation zone boundaries, the 2D TDE maps were rigidly deformed to the intraoperative MRIs and visualized together with slices of the manually-traced ablation volumes through the same imaging plane as the TDE maps. Pixel overlap between two regions (A and B) aligned in the same imaging cut was calculated using the dice similarity coefficient (DSC):

$$DSC(A,B)=\frac{2(A\cap B)}{A+B}$$

which ranges from 0 to 1, where 0 indicates no overlap and 1 indicates complete overlap [12, 22, 27].

Composite ablation maps were calculated by aligning TDE maps in a straight line along the laser fiber and then rigidly deforming these 2D areas along this axis so that they lined up end to end. Similar deformations were applied to manually-traced immediate and delayed ablation boundaries through the same imaging plane as the TDE map. The distance from the laser fiber to boundaries of the TDE and postoperative ablation zones was then measured perpendicular to the laser catheter, along 20 equidistant points, in millimeters (since maps were not normalized in this axis). A ruler contained in the Visualase workstation was used to confirm pixel to millimeter conversions for each patient’s TDE maps.

Time series of irreversible ablation were calculated from the TDE videos (each frame typically 6.7 seconds). For each pixel we determined whether irreversible damage occurred (i.e. if pixel turned yellow) and, if so, when after ablation onset this happened and location of the pixel relative to the laser fiber. These pixels were measured either for the entire ablation zone or for predefined regions, such as superior/inferior or mesial/lateral to the laser (Fig 1G and 1H). To remove confounding effects of prior ablations, only the first ablations (after the 30% test dose) were used for analyses, which always involved amygdala and hippocampal head.

### Statistical analyses

Matlab was used for statistical analyses. All comparisons of means were performed using bootstrap analyses with data resampled 1000 times and p < 0.05 used as the criterion for significance. Ablation dynamics were modeled by fitting pixel count vs. time from the TDE videos with an exponential curve:

$$TDE(t)=C(1-e^{-\tau^{-1}(t-t_{shift})}$$

with *τ* the time constant, *C* the exponential coefficient specifying the maximum number of TDE pixels ablated for large values of *t*, and *t*_*shift*_ the time shift. For our correlation analyses the inverse time constant (τ^-1^) was used since this showed more consistent linear correlations with the explanatory variables. To assess factors mediating mesial vs. lateral extent of ablation, we formed the difference between the time series of mesial TDE pixels minus lateral TDE pixels. The same was also done for superior minus inferior pixels. Since the temporal dynamics of this difference were governed by similar exponential dynamics, these data were divided into early (before 60 seconds) and late phases (after 60 seconds), which were fit with independent linear regression models:

$$TDE_{medial}(t)-TDE_{lateral}(t)=m_{ML}\cdot t+b_{ML}$$

with *m*_*ML*_ (or *m*_*SI*_) the slope and *b*_*ML*_ (or *b*_*SI*_) the intercept.

Sixteen independent variables were determined for each patient: 1) age, 2) sex, 3) presence of mesiotemporal sclerosis (MTS), 4) CSF_Above_, 5) CSF_Lateral_, 6) AHC volume, 7) T1 spin echo signal, 8) T1 gadolinium (GAD) signal, 9) T2 fat suppression signal, 10) total joules, 11) ablation time, 12) mean laser power, 13) laser medial-lateral (M-L) position, 14) laser superior-inferior (S-I) position, 15) axial angle and 16) sagittal angle. Univariate and multivariate linear regression models for the dependent variables *τ* ^*-1*^, *C*, *t*_*shift*_, *m*_*ML*_, *b*_*ML*_, *m*_*SI*_, *b*_*SI*_ were then explored using these independent variables.

## Results

Of 30 patients with over 6-months follow-up included in this study, sixteen (53%) were male. Average age (± SD) was 43.5 ± 11.3 years. Twenty-one (70%) had evidence of MTS. Average duration from surgery to acquisition of postoperative MRI was 6.4 ± 3.2 months. All underlying demographic and imaging data used are shown in S1 and S2 Tables.

### Relation of TDE maps to ablation boundaries

We expected that as a result of secondary involutional changes post-LITT [20, 21], delayed 6-month ablation volumes would assume a different size and shape than immediate postoperative ablation volumes. Indeed, while immediate ablations were generally in the same location as the delayed ablations, they were consistently larger (5,478 ± 1,195 mm^3^ vs. 2,440 ± 759 mm^3^, p < 0.001, bootstrap). Such two-fold differences in acute vs. chronic lesion volumes have been observed for brain tumors after LiTT [20, 21, 24].

**Fig 2:**
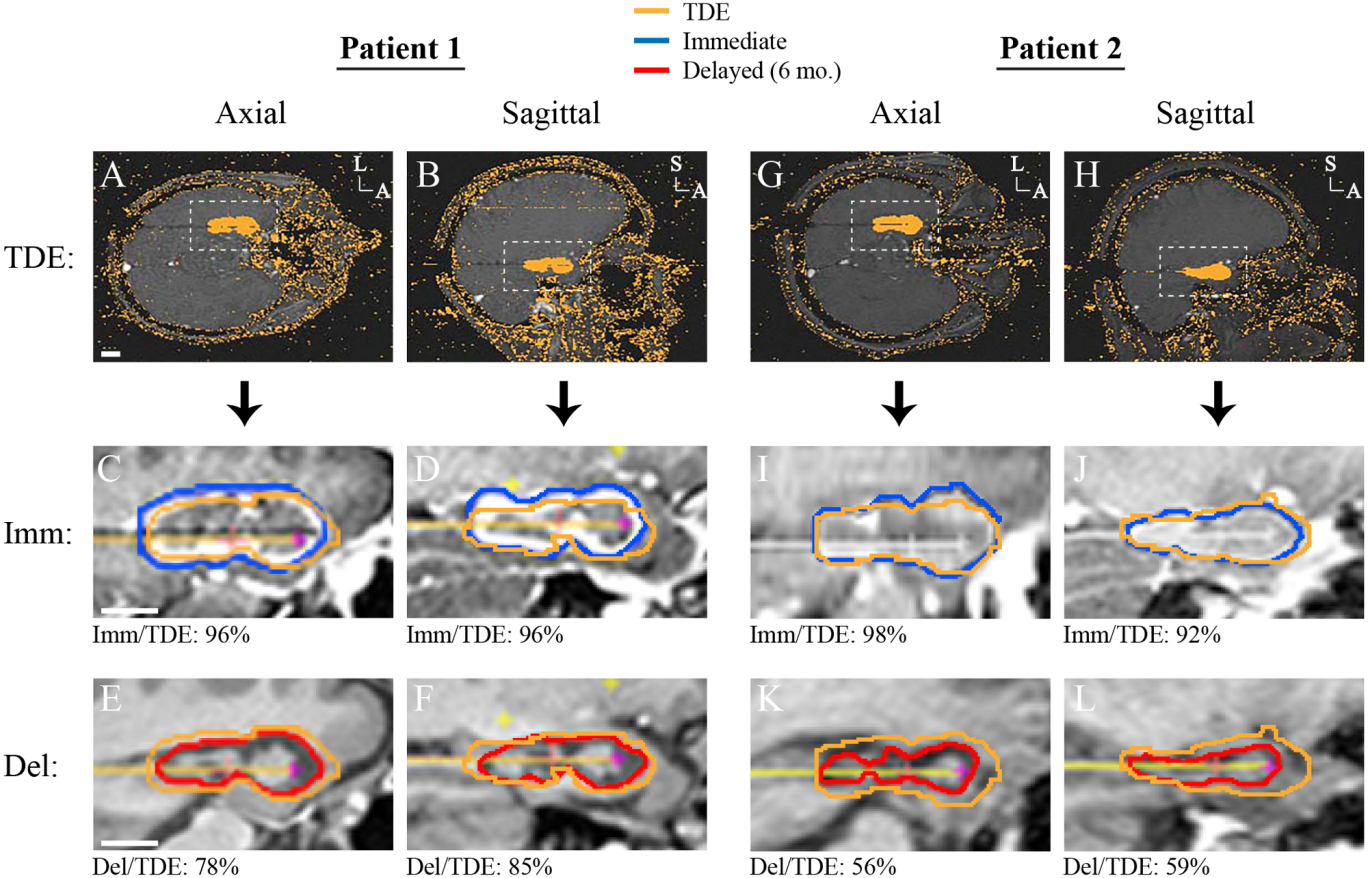
Overlap of TDE maps with immediate and delayed ablation zone boundaries. (A-B) Axial and sagittal TDE maps at the end of the LITT procedure for sample Patient 1. The white dashed boxes show the regions highlighted in the panels below. (C-D) Axial and sagittal post ablation contrasted T1 images showing the manually-traced immediate ablation zone (blue) and the boundary of the TDE (yellow) from the same imaging plane. The numbers below the image panels show the DSC values. (E-F) Axial and sagittal T1 MP-RAGE images 6-months after surgery demonstrating the manually-traced delayed ablations (red) and the TDE (yellow). (G-L) Same conventions as A—F but for Patient 2. A, anterior; Del, delayed; Imm, immediate; L, lateral; S, superior; TDE, thermal damage estimate.

Intraoperative TDE maps closely resembled immediate ablation zones but were smaller (Fig 2, middle panel). The axial TDEs were 13% smaller in cross-sectional area than immediate ablation zones through the same plane (493 ± 81 mm^2^ vs. 565 ± 97 mm^2^, p < 0.001). The sagittal TDEs were 20% smaller in area than the immediate ablations (384 ± 62 mm^2^ vs. 479 ± 103 mm^2^, p < 0.001, bootstrap). The overlap of immediate ablation zone boundaries with TDE maps, measured as DSC(Immediate, TDE), was 0.92 ± 0.06 in the axial plane and 0.94 ± 0.04 in the sagittal plane, consistent with prior studies [9, 12, 28]. No significant associations were identified for DSC(Immediate, TDE) and the 16 independent variables.

In contrast, when delayed ablation zones were compared to the TDE maps greater discrepancies in size and shape were observed (Fig 2, bottom panel). For several patients the delayed ablation cavity nearly matched the TDE, but for the majority they were smaller and of different shape. The TDEs were 28% larger than the delayed ablation zones in the axial plane (495 ± 81 mm^2^ vs. 386 ± 86 mm^2^, p < 0.001) and 14% larger in the sagittal plane (386 ± 61 mm^2^ vs. 337 ± 70 mm^2^, p = 0.03). DSC(Delayed, TDE) was 0.74 ± 0.11 in the axial plane and 0.82 ± 0.09 in the sagittal plane. When univariate regression analysis was applied to examine the relationship of axial DSCs to the 16 independent variables, DSC(Delayed_Axial_, TDE_Axial_) was negatively correlated with CSF_Above_ (F_1_ = 4.0, R^2^ = 0.13, p = 0.05). Multivariate analysis using all 16 independent variables failed to achieve statistical significance (F_16_ = 1.88, R^2^ = 0.71, p = 0.13), but was suggestive of associations between DSC(Delayed_Axial_, TDE_Axial_) and gender, CSF above, AHC volume, T1 and T2 signal (Table 1). For the sagittal TDE maps, the only association noted was a positive trend for DSC(Delayed_Sagittal_, TDE_Sagittal_) with T2 signal (F_1_ = 4.1, R^2^ = 0.20, p = 0.06).

**Table 1:** Regression coefficients for independent variables with the DSC(Delayed_Ax_, TDE_Ax_).

Variable	DSC(Delayed_Ax_, TDE_Ax_)	
	Coef.	P
**Age**	- 0.003	0.14
**Gender**	**- 0.122**	**0.04**
**Presence of MTS**	- 0.065	0.35
**CSF above**	**- 0.001**	**0.05**
**CSF lateral**	- 0.002	0.36
**AHC volume**	**0.000**	**0.03**
**T1 signal**	**- 0.000**	**0.05**
**T1 GAD signal**	0.000	0.19
**T2 signal**	**0.001**	**0.02**
**Ablation energy**	- 0.000	0.41
**Ablation time**	0.001	0.38
**Ablation power**	0.027	0.77
**M-L position**	- 0.480	0.12
**S-I position**	- 0.179	0.41
**Axial angle**	- 0.009	0.49
**Sagittal angle**	0.000	0.94

Multivariate regression analyses of DSC overlap values for delayed (6 mo.) ablation zones with axial TDE maps. Shown are regression coefficients and p-values for the individual variables calculated from the multivariate analyses. Measures with p ≤ 0.05 are bolded. AHC, amygdalohippocampal cortex; GAD, gadolinium; MTS, mesiotemporal sclerosis; M-L, mesial-lateral; S-I, superior-inferior.

**Fig 3:**
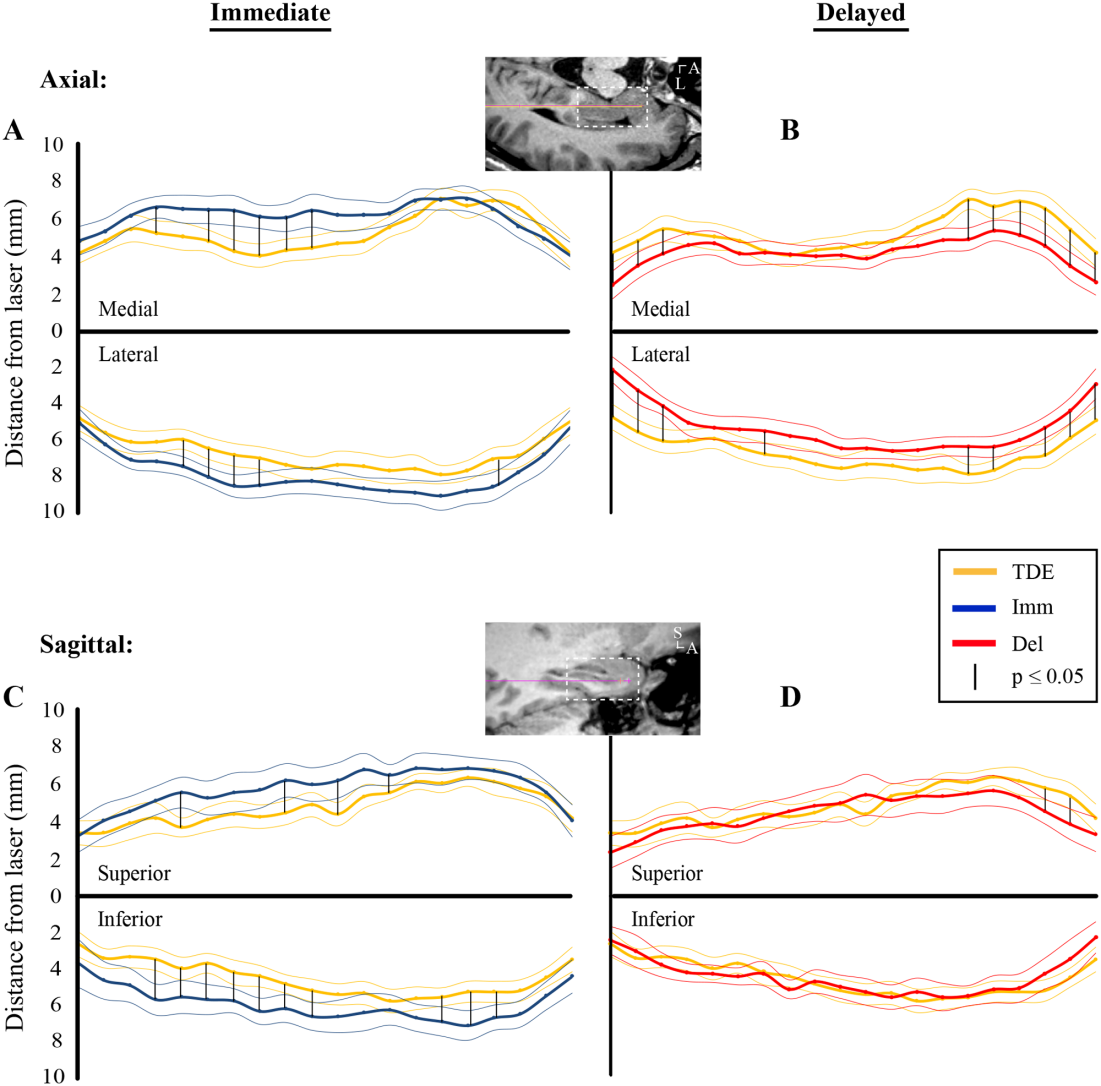
Comparison of averaged TDE maps and ablation zone boundaries for entire patient cohort. (A) Composite maps showing averaged axial TDEs (yellow) and immediate ablation boundaries through the same imaging plane (blue) for all patients in the study (N = 28). Thick lines show mean distance from the laser and thin lines mean ± standard error. Vertical black lines show positions along the laser where TDE maps differed from the ablation zones with p ≤ 0.05, determined using bootstrap analysis. (B) Axial TDE maps (yellow) and delayed ablation boundaries (red). The axial MRI above estimates the mesiotemporal location of data used for A and B. (C) Sagittal TDE maps (yellow) and immediate ablation boundaries (blue) (N = 16). (D) Sagittal TDE maps (yellow) and delayed ablation boundaries (red). The sagittal MRI above estimates the mesiotemporal location of data used for C and D. A, anterior; Del, delayed; Imm, immediate; L, lateral; S, superior; TDE, thermal damage estimate.

To determine whether differences in postoperative ablation zone boundaries and TDEs vary by anatomic region we next normalized and averaged all TDE maps and associated ablation zones. Fig 3A shows that axial TDEs better approximate immediate ablation zone boundaries at their anterior and posterior ends but less so in the middle, at the hippocampal body. The delayed ablation cavities (Fig 3B), however, were more closely approximated by the TDE maps in the middle of the lesions, with the most anteromedial aspects of the ablations, corresponding to mesial portions of amygdala and hippocampal head, showing greatest deviation from the TDEs. A similar trend was observed for the sagittal maps (Fig 3C and 3D), but the only significant differences observed for delayed ablation zones and TDE maps were anterosuperiorly, at the amygdala.

### Intraoperative ablation dynamics

#### Total pixel counts

**Fig 4:**
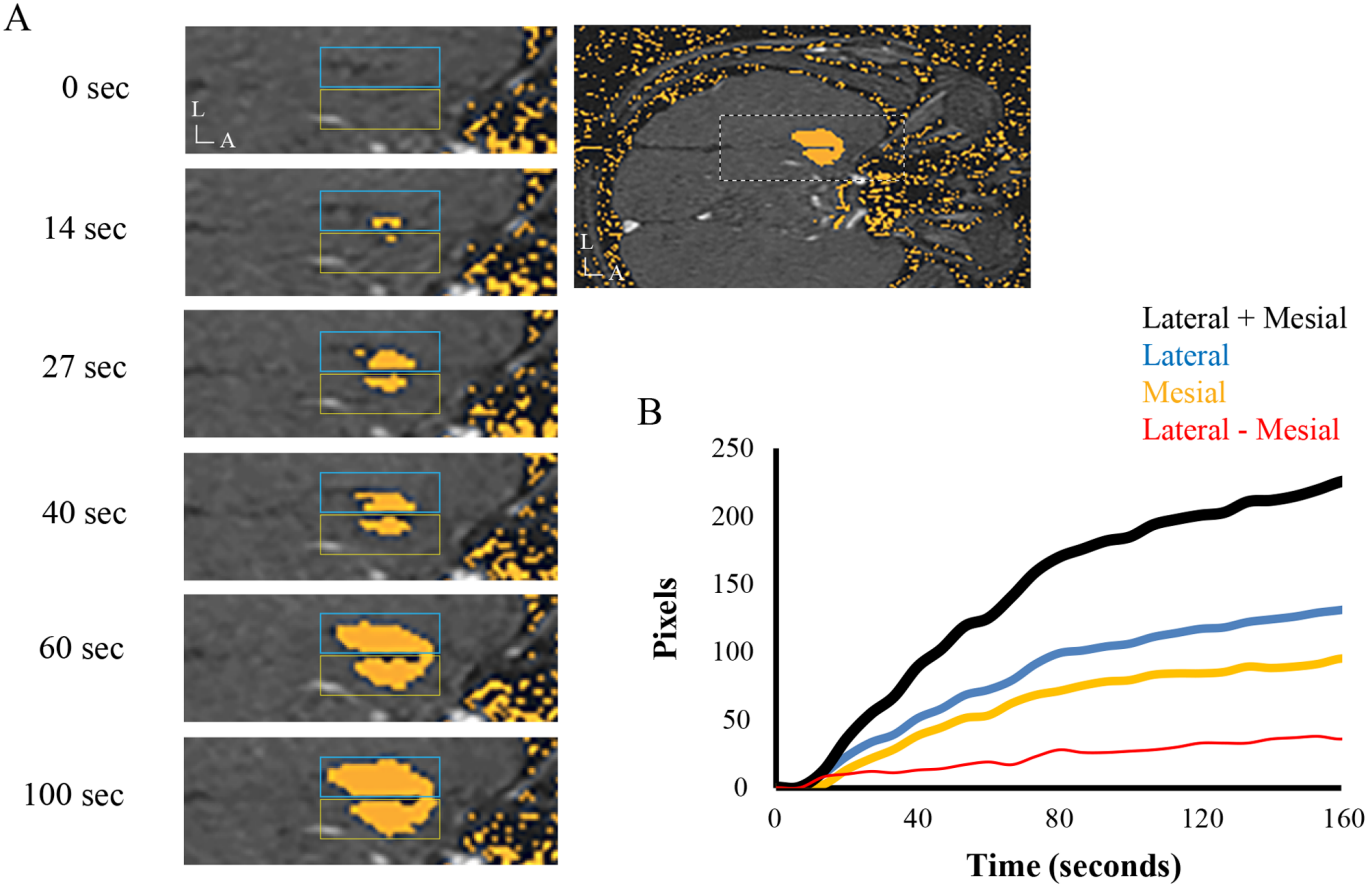
Dynamics of irreversible ablation during mesiotemporal LITT. (A) Different frames of a sample axial TDE video. The larger image on the right shows a lower magnification view, with the white dashed box highlighting the region used in the left panels. Trajectories were traced from the first frame in each video and the appearance of yellow pixels indicating irreversible ablation was quantified relative to the laser using Matlab Image Processing Toolbox. The blue (lateral) and yellow (mesial) rectangles demonstrate regions of interest used to measure pixel counts on each side of the laser. (B) Time course of total (black), lateral (blue), mesial (yellow), and lateral—mesial (red) pixel counts from the same patient as in G. A, anterior; L, lateral.

Soon after laser activation, the number of pixels representing irreversibly-ablated tissue sharply increases, typically reaching a plateau after 2–3 minutes (Fig 4). Pixel counts, which were well-modeled by exponential curves (axial median [range] R^2^ = 0.992 [0.961, 0.998], sagittal median R^2^ = 0.989 [0.885, 0.997]), showed variable responses to laser energy. For axial and sagittal TDE maps, respectively, mean (± SD) time constant, *τ*, was 92 ± 61 seconds and 108 ± 118 seconds, exponential coefficient, *C*, was 284 ± 75 and 120 ± 33, and time shift, *t*_*shift*_, was 0.7 ± 5.7 seconds and 2.6 ± 3.4 seconds.

Results of uni- and multi-variate regression analyses are shown in Table 2. For the axial TDEs, univariate analysis suggested a negative correlation between *τ* ^*-1*^ and MRI T2 signal, implying greater T2 signal is associated with slower ablation. Trajectories with more mesial-pointing laser catheters in the axial plane also were correlated with slower ablation. Results of multivariate analyses examining the relationship of *τ* ^*-1*^ to all independent variables is shown in Table 3. For the sagittal TDE maps, slower ablation (low *τ* ^*-1*^) was correlated with high laser power and laser position near the choroidal fissure on the D-V axis using univariate analyses. Non-significant trends were observed for AHC volume (positive, R^2^ = 0.19, P = 0.09), T1fe GAD signal (negative, R^2^ = 0.13, P = 0.16), and T2 signal (negative, R^2^ = 0.12, P = 0.18) with sagittal *τ*^*-1*^.

**Table 2:** Relationship of ablation dynamics measures to the independent variables.

	Significant univariate regressors			Multivariate model
**Axial**				
*τ* ^*-1*^	**T2 signal**	**Axial angle**		R^2^ = 0.44
	–	–		P = 0.003
	P = 0.009	P = 0.04		
*C*	NS			
*t*_*shift*_	**Sagittal angle**	**Mean laser power**	**CSF lateral**	R^2^ = 0.34
	+	–	+	P = 0.02
	P = 0.01	P = 0.04	P = 0.05	
**Sagittal**				
*τ* ^*-1*^	**S-I position**	**Mean laser power**		R^2^ = 0.37
	+	–		P = 0.05
	P = 0.02	P = 0.04		
*C*	NS			
*t*_*shift*_	NS			

Shown in the middle column are independent variables that achieved statistical significance when univariate regression analyses were used to identify associations with the ablation dynamics measure shown in the left column. Below each independent variable is the sign of the correlation and P value. In the right column, if a multivariate model using the same variables achieves statistical significance, the R^2^ and P values of the analysis are listed. *C*, exponential coefficient; *τ* ^*-1*^, inverse time constant; NS, not statistically significant; *t*_*shift*_, time shift.

**Table 3:** Regression coefficients for independent variables with the thermal time constant.

Variable	*τ* ^*-1*^	
	Coef.	P
**Age**	0.000	0.67
**Gender**	- 0.001	0.48
**Presence of MTS**	0.000	0.85
**CSF above**	0.000	0.63
**CSF lateral**	- 0.001	0.23
**AHC volume**	0.000	0.67
**T1 signal**	0.000	0.95
**T1 GAD signal**	- 0.001	0.10
**T2 signal**	**- 0.002**	**0.01**
**Ablation energy**	- 0.002	0.08
**Ablation time**	- 0.001	0.14
**Ablation power**	- 0.001	0.37
**M-L position**	0.001	0.23
**S-I position**	- 0.001	0.35
**Axial angle**	**- 0.002**	**0.04**
**Sagittal angle**	0.001	0.16

Shown are univariate regression analyses for the 16 independent variables and τ^-1^ calculated from the axial TDE videos. For each variable, the estimated regression coefficient and p-value are shown. Measures with p ≤ 0.05 are bolded. AHC, amygdalohippocampal cortex; GAD, gadolinium; MTS, mesiotemporal sclerosis; M-L, mesial-lateral; S-I, superior-inferior.

In the axial plane, *t*_*shift*_ was correlated positively with sagittal angle and CSF_Lateral_ and negatively with mean laser power (Table 2). In contrast, no associations were identified for *t*_*shift*_ from the sagittal TDE maps with the independent variables. Furthermore, no relationships were identified for the ablation exponential coefficients, *C*, with the independent variables in either imaging plane (See S3 Table for additional results of multivariate analyses).

#### Ablation symmetry

**Fig 5:**
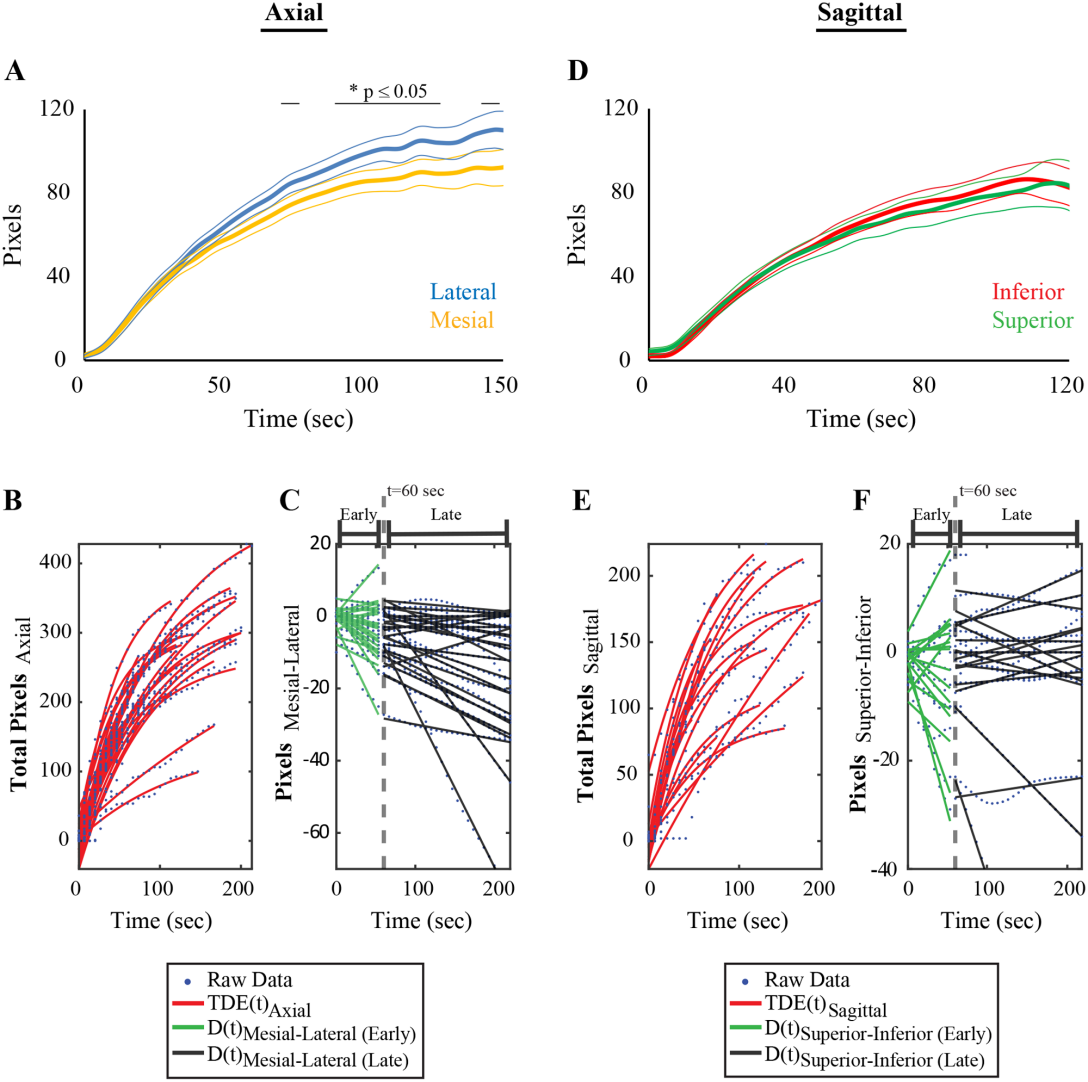
Mesiotemporal LITT ablation asymmetry. (A) Time course of pixel counts lateral (blue) and mesial (yellow) to the laser averaged from all axial TDE maps (N = 29). Pixel counts were greater lateral vs. mesial to the laser past 60 seconds, with statistically significant differences between the curves shown with a horizontal line above the graph. At each time point, significance was evaluated with bootstrap analysis using p ≤ 0.05 as the criterion. (B) Pixel counts measured from the axial TDE videos fit with exponential curves for each patient (N = 28). (C) Difference in mesial and lateral pixel counts modeled with best-fit lines for each patient, done separately for early and late differences, distinguished by the 60 second time point (dotted grey line). (D-F) Same conventions as A—C but calculated from sagittal TDE maps (N = 16). Unlike for the axial maps, no differences were found for pixel counts measured superior (green) vs. inferior (red) to the laser.

Clinical experience had suggested asymmetry in the mesiolateral and superoinferior progression of LITT ablations. When pixel counts on either side of the laser were compared for all patients, degree of ablation was higher in lateral vs. mesial directions for the axial TDE maps, which reached statistical significance past 60 seconds (Fig 5). No differences in ablation were noted superior vs. inferior to the laser for sagittal TDE maps.

To elucidate factors associated with ablation symmetry we quantified the dynamics of pixel count differences across the laser fiber and examined their relationship to the independent variables. For early (before 60 seconds) and late (after 60 seconds) ablations, dynamics of ablation asymmetry were quantified using D(t)_Mesial-Lateral_ = TDE_Mesial_(t)–TDE_Lateral_(t), which was modeled linearly (Fig 5B and 5C). For axial TDE maps, regression analysis found that during early ablation greater lateral spread was associated with a more superior trajectory, greater angle in the sagittal plane, and higher T1 and T1 GAD signal at the target (Table 4, top panel). Late in the ablation greater lateral spread was correlated with higher sagittal angle. For sagittal TDE maps, a higher rate of superior vs. inferior spread was found late in the ablations for patients with greater mesiotemporal T2 signal and T1 GAD signal (Table 4, lower panel).

**Table 4:** Evaluation of ablation asymmetry.

	Significant univariate regressors		Multivariate model
**Axial**			
Early *m*_*ML*_	**S-I position**	**Sagittal angle**	R^2^ = 0.30
	+	–	P = 0.01
	P = 0.02	P = 0.04	
Early *b*_*ML*_	**T1 GAD signal**	**T1 signal**	
	–	–	NS
	P = 0.02	P = 0.02	
Late *m*_*ML*_	NS		
Late *b*_*ML*_	**Sagittal angle**		
	–		
	P = 0.01		
**Sagittal**			
Early *m*_*SI*_	NS		
Early *b*_*SI*_	NS		
Late *m*_*SI*_	**T2 signal**	**T1 GAD signal**	
	+	+	NS
	P = 0.04	P = 0.05	
Late *b*_*SI*_	NS		

The middle column shows independent variables that achieved statistical significance when univariate regression analyses were used to identify associations with ablation symmetry dynamics measured from best-fit lines (left column). Below each independent variable is the sign of the correlation and P value. Because we are forming a difference in pixel counts, a (+) could indicated either increased mesial (or superior) or decreased lateral (or inferior) ablation. If a multivariate model using the same variables achieved statistical significance, the R^2^ and P values of the analysis are shown in the right column. *C*, exponential coefficient; *τ* ^*-1*^, inverse time constant; NS, not statistically significant; *t*_*shift*_, time shift. *b*, intercept; *m*, slope; ML, mesial—lateral; NS, not statistically significant; SI, Superior—Inferior.

## Discussion

Here, we performed an extensive analysis of Arrhenius-derived TDE maps acquired during LITT of mesiotemporal structures in an attempt to define variables that influence the irreversible coagulation of brain tissue. While these findings are preliminary, owing to the fact that numerous independent variables were analyzed for a small and diverse patient population, we do believe the mesiotemporal model utilized here is the ideal model currently available for studying LITT ablations in humans. Because several variables from MRI, such as T2 signal, volume of surrounding CSF, and laser trajectory, were consistently shown by our analyses to be associated with the short- and long-term progression of LITT ablations, this does imply that the accuracy with which LITT is planned and delivered could one day be improved by incorporating imaging data into predictive models of LITT effects.

### Not all mesiotemporal tissues ablate equally

During LITT the penetration of photons into the brain is limited, particularly as tissue coagulates and optical properties change. Convective spread of heat thus becomes an important mechanism of lesion progression [11, 19]. Since the earliest reports on LITT, features of local anatomy relevant to convection have been presumed to be important determinants of tissue thermoablative properties. Blood vessels and CSF spaces are hypothesized to act as heat sinks that dampen the thermal response and limit the spatial extent of lesions [11–13, 17, 19]. Cisterns and sulci may act as physical barriers and reflectors of light that also limit heat spread [1, 17, 19]. Elucidating these factors and their impact on LITT is important for mesiotemporal epilepsy, since amygdalohippocampal anatomy varies tremendously, with differing degrees of scarring, atrophy, and CSF dilatations and cysts observed between patients [6, 16, 25]. Here, although our sample size is small and results are not consistent between axial and sagittal maps, data do suggest an influence of preoperative anatomy on the progression of the ablations.

Greater laser energy appears to be needed for coagulation of tissues with high water content or close to large CSF spaces. High laser power was negatively correlated with *t*_*shift*_, indicating faster ablation onset with higher laser power, as we had expected. However, the negative correlation of laser power with *τ* ^*-1*^, implying slower ablations with greater power was counter intuitive. One possible explanation is use of lower laser power by the surgeon for patients with quick temperature increases during the test dose. Other potential causes include an unknown confounder or, alternatively, an impediment to optical transfer of heat to nearby tissues at higher temperatures, thus making the ablation slower. However, deeper investigation of such processes is not possible using our study paradigm. Furthermore, from our ablation symmetry data it is also not possible to conclude whether greater laser energy is required to coagulate mesial vs. lateral regions of the target or if this was an artifact of positioning the laser too close to a border or CSF space. If in fact mesial amygdala and hippocampal head do have higher ablation thresholds, we speculate it to be the result of proximity to the large ambient cistern and possible convective heat loss due to larger cisternal vessels.

TDE maps are well-correlated with ablation boundaries measured from immediate postoperative imaging, but less so with lesions measured several months after surgery, when edema and mass effect have subsided and scarring has begun. This is not surprising since immediate postoperative imaging is typically used to evaluate the predictive models, with little attention paid to delayed structural changes [12, 28]. Qualitative differences in TDE maps and final ablation zone boundaries have been reported before. Atsina et al. [20] in a retrospective analysis of 23 epilepsy patients observed that while TDE maps are generally predictive of extent of ablation as determined from immediate postoperative MRI, in patients with follow-up imaging eight showed a decreased extent of ablation with follow-up, two showed no change in size, and one patient (with less follow-up, 7 days) showed a greater extent of ablation than predicted by the TDE maps.

The finding that TDE maps overestimate lesion size relative to delayed imaging could have been due to early-onset swelling during TDE acquisition that inflated predictions of ablation extent, or to lesion contraction in the months after surgery that caused an underestimation of LITT effects [20, 29]. These factors could also account for the discrepancy between TDE maps and delayed ablation effects at amygdala and hippocampal head since this is always the sub-region of the ablation target that receives the greatest thermal dose, making it the most likely area to show treatment-related effects of tissue swelling or contraction on the DSC measures. Yet another possibility is that due to the uncertainty in calculating TDE maps, the fates of tissues at the periphery of the lesions may be difficult to predict. Tissues marked as irreversibly ablated may still survive, or vice versa. Early literature on LITT did suggest the possibility of a grey zone at the TDE periphery [10, 18]. Whether such a region at the cuff of the ablation exists is unknown and beyond the scope of this work. However, if so, then we certainly expect the proposed heat-dissipating properties of blood vessels or of tissue edema to influence the survival of cells in this location, which could in part explain the covariance of our delayed DSC values with CSF volume and T2 signal. We attempted to evaluate this further by comparing “non-ablated” mesial hippocampal head tissue from immediate vs. delayed imaging, but this wasn’t possible from immediate postoperative imaging given the degree of swelling at the lesion and distortion of surrounding anatomy.

### Modelling LITT ablations

Understanding tissue thermoablative properties and constraints imposed by local anatomy could aid in preoperative treatment planning. Tissues at the periphery of the target or with high ablation thresholds may require unique trajectories or ablation parameters. Alternatively, a second laser catheter may be necessary [1, 15]. By estimating tissue thermal responses *a priori* from preoperative imaging, it should be possible to improve outcomes by reducing frequency of missed ablations. An example is the mesial hippocampal head, which has been suggested to have an important role in seizure control, though further investigation is necessary to confirm this [4]. Our observation that greater laser energy may be required to ablate mesial versus lateral mesiotemporal structures could reinforce the need for a mesial trajectory to maximize likelihood of achieving seizure freedom. The preoperative estimation of tissue thermoablative properties will likely become more important as we continue to elucidate mesiotemporal sub-regions critical to treating seizures and preserving neurocognitive function, hence demanding greater spatial precision of the technique.

We caution that our use of arbitrary independent variables is likely not the best means for capturing the variance in thermoablative properties. A bottom-up physics-centered approach considering tissue optical properties may have been better at modeling these processes [11, 19]. However, such an approach requires detailed understanding of tissue specific properties to a degree that is largely absent for the brain and unavailable for use in the standard clinical setting. Nevertheless, the procedures described here allow us to infer ablation dynamics for different disease states and may eventually help build a bottom-up approach using insights gained from these approaches. Another limitation of this study is the changes in mesiotemporal anatomy caused by edema and swelling soon after ablation onset and by tissue contraction in the months after surgery, which certainly influenced quality of image co-registration [20, 29]. However, we believe our use of the deformable atlas reduced the likelihood of improper alignment. In addition, the rate of fluid flow through the laser was not controlled for and while this could affect tissue heating, similar flow settings were used for the entire cohort and thus likely did not impact results of the study. Also, predictions based on the Arrhenius equation require adequate knowledge of temperature, which is derived from MRI thermography [7, 30, 31]. Errors in temperature measurement or changes in patient temperature during an ablation could affect MRI-based temperature estimates and impact predictions of ablation extent. Lastly, we were unable to measure tissue perfusion, which is known to have a strong influence on tissue thermal ablation [11, 17, 19].

In order for LITT to reach its full potential with respect to established open surgical therapies it is important that all potentially beneficial data are incorporated into our models. Elucidating the factors that influence LITT ablations will not only require a greater number of cases, but also development of advanced image analysis tools capable of normalizing the data generated and identifying correlates of good responses. With such information, we could eventually create population-based statistical atlases capable of predicting tissue thermoablative properties with high anatomical resolution for individual patients. Not only could such aggregate data improve our selection of patients eligible for treatment, but it could also be used to plan optimal trajectories, estimate laser settings required for safe coagulation of the target, and then modify these estimates in real-time as feedback from intraoperative imaging is acquired [8, 32]. In addition, availability of normative cross-patient thermoablative properties would put us one step closer to being able to safely offer LITT without the need for real-time MRI monitoring of the ablation. In well-selected patients this could significantly decrease procedure duration without sacrificing safety, thereby reducing patient discomfort and risks of anesthesia and freeing up valuable hospital resources [15, 33].

## Supporting information

S1 Table Demographic, LITT ablation dynamics, DSC overlap and independent variable data for axial TDE videos. N = 29. AHC, amygdalohippocampal cortex; GAD, gadolinium; MTS, mesiotemporal sclerosis; M-L, mesial-lateral; S-I, superior-inferior.
(XLSX)

S2 Table Demographic, LITT ablation dynamics, DSC overlap and independent variable data for sagittal TDE videos. N = 17. AHC, amygdalohippocampal cortex; GAD, gadolinium; MTS, mesiotemporal sclerosis; M-L, mesial-lateral; S-I, superior-inferior.
(XLSX)

S3 Table Regression coefficients of independent variables with the thermal modeling parameters. Shown are univariate regression analyses for the 16 independent variables and τ^-1^ calculated from the axial TDE videos. For each variable, regression coefficient and p-value are shown. Measures with p ≤ 0.05 are bolded. AHC, amygdalohippocampal cortex; GAD, gadolinium; MTS, mesiotemporal sclerosis; M-L, mesial-lateral; S-I, superior-inferior.
(DOCX)

## References

[1] Willie JT, Laxpati NG, Drane DL, Gowda A, Appin C, Hao C, et al Real-time magnetic resonance-guided stereotactic laser amygdalohippocampotomy for mesial temporal lobe epilepsy. Neurosurgery. 2014;74(6):569–84; discussion 84–5. doi: 10.1227/NEU.0000000000000343 24618797 10.1227/NEU.0000000000000343 PMC4151501

[2] Waseem H, Osborn KE, Schoenberg MR, Kelley V, Bozorg A, Cabello D, et al Laser ablation therapy: An alternative treatment for medically resistant mesial temporal lobe epilepsy after age 50. Epilepsy Behav. 2015;51:152–7. doi: 10.1016/j.yebeh.2015.07.022 26280814 10.1016/j.yebeh.2015.07.022

[3] Kang JY, Wu C, Tracy J, Lorenzo M, Evans J, Nei M, et al Laser interstitial thermal therapy for medically intractable mesial temporal lobe epilepsy. Epilepsia. 2016;57(2):325–34. doi: 10.1111/epi.13284 26697969 10.1111/epi.13284

[4] Jermakowicz WJ, Kanner AM, Sur S, Bermudez C, D’Haese PF, Kolcun JPG, et al Laser thermal ablation for mesiotemporal epilepsy: Analysis of ablation volumes and trajectories. Epilepsia. 2017;58(5):801–10. doi: 10.1111/epi.13715 28244590 10.1111/epi.13715 PMC5429877

[5] Drane DL, Loring DW, Voets NL, Price M, Ojemann JG, Willie JT, et al Better object recognition and naming outcome with MRI-guided stereotactic laser amygdalohippocampotomy for temporal lobe epilepsy. Epilepsia. 2015;56(1):101–13. doi: 10.1111/epi.12860 25489630 10.1111/epi.12860 PMC4446987

[6] Jermakowicz WJ, I M, Cajigas I, Ribot R, Jusue-Torres I, Desai MB, Ruiz A, D’Haese PF, Kanner AM, Jagid JR. Visual Deficit From Laser Interstitial Thermal Therapy for Temporal Lobe Epilepsy: Anatomical Considerations. Operative Neurosurgery. 2017;opx029. 10.1093/ons/opx029 PMC5606145 28922876

[7] Breen MS, Breen M, Butts K, Chen L, Saidel GM, Wilson DL. MRI-guided thermal ablation therapy: model and parameter estimates to predict cell death from MR thermometry images. Ann Biomed Eng. 2007;35(8):1391–403. doi: 10.1007/s10439-007-9300-3 17436111 10.1007/s10439-007-9300-3

[8] Lehmann KS, Frericks BB, Holmer C, Schenk A, Weihusen A, Knappe V, et al In vivo validation of a therapy planning system for laser-induced thermotherapy (LITT) of liver malignancies. Int J Colorectal Dis. 2011;26(6):799–808. doi: 10.1007/s00384-011-1175-y 21404055 10.1007/s00384-011-1175-y

[9] Patel NV, Frenchu K, Danish SF. Does the Thermal Damage Estimate Correlate With the Magnetic Resonance Imaging Predicted Ablation Size After Laser Interstitial Thermal Therapy? Oper Neurosurg (Hagerstown). 2017. 10.1093/ons/opx191 28962036

[10] Pearce JA. Improving Accuracy in Arrhenius Models of Cell Death: Adding a Temperature-Dependent Time Delay. J Biomech Eng. 2015;137(12):121006 doi: 10.1115/1.4031851 26501738 10.1115/1.4031851

[11] Saccomandi P, Schena E, Di Matteo FM, Pandolfi M, Martino M, Rea R, et al Theoretical assessment of principal factors influencing laser interstitial thermotherapy outcomes on pancreas. Conf Proc IEEE Eng Med Biol Soc. 2012;2012:5687–90. doi: 10.1109/EMBC.2012.6347286 23367221 10.1109/EMBC.2012.6347286

[12] Yung JP, Shetty A, Elliott A, Weinberg JS, McNichols RJ, Gowda A, et al Quantitative comparison of thermal dose models in normal canine brain. Med Phys. 2010;37(10):5313–21. doi: 10.1118/1.3490085 21089766 10.1118/1.3490085 PMC2955727

[13] Sun XR, Patel NV, Danish SF. Tissue Ablation Dynamics During Magnetic Resonance-Guided, Laser-Induced Thermal Therapy. Neurosurgery. 2015;77(1):51–8; discussion 8. doi: 10.1227/NEU.0000000000000732 26086908 10.1227/NEU.0000000000000732

[14] Wong T, Patel NV, Feiteiro F, Danish SF, Hanft S. Lesion Optimization for Laser Ablation: Fluid Evacuation Prior to Laser-Induced Thermal Therapy. World Neurosurg. 2017;104:192–6. doi: 10.1016/j.wneu.2017.04.167 28479523 10.1016/j.wneu.2017.04.167

[15] Kamath AA, Friedman DD, Hacker CD, Smyth MD, Limbrick DD Jr., Kim AH, et al MRI-Guided Interstitial Laser Ablation for Intracranial Lesions: A Large Single-Institution Experience of 133 Cases. Stereotact Funct Neurosurg. 2018;95(6):417–28. 10.1159/000485387 29339639

[16] Zachenhofer I, Novak K, Baumgartner C, Prayer D, Czech T. Reoperation after selective amygdalohippocampectomy: an MRI analysis of the extent of temporomesial resection in ten cases. Acta Neurochir (Wien). 2011;153(2):239–48. 20853122 10.1007/s00701-010-0802-7

[17] Saccomandi P, Schena E, Caponero MA, Di Matteo FM, Martino M, Pandolfi M, et al Theoretical analysis and experimental evaluation of laser-induced interstitial thermotherapy in ex vivo porcine pancreas. IEEE Trans Biomed Eng. 2012;59(10):2958–64. doi: 10.1109/TBME.2012.2210895 22929361 10.1109/TBME.2012.2210895

[18] Whelan WM, Wyman DR. Dynamic modeling of interstitial laser photocoagulation: implications for lesion formation in liver in vivo. Lasers Surg Med. 1999;24(3):202–8. 10229151 10.1002/(sici)1096-9101(1999)24:3<202::aid-lsm5>3.0.co;2-8

[19] Zhu D, Luo Q, Zhu G, Liu W. Kinetic thermal response and damage in laser coagulation of tissue. Lasers Surg Med. 2002;31(5):313–21. doi: 10.1002/lsm.10108 12430148 10.1002/lsm.10108

[20] Atsina KB, Sharan AD, Wu C, Evans JJ, Sperling MR, Skidmore CT, et al JOURNAL CLUB: Longitudinal Qualitative Characterization of MRI Features After Laser Interstitial Thermal Therapy in Drug-Resistant Epilepsy. AJR Am J Roentgenol. 2017;208(1):48–56. doi: 10.2214/AJR.16.16144 27657929 10.2214/AJR.16.16144

[21] Patel NV, Jethwa PR, Barrese JC, Hargreaves EL, Danish SF. Volumetric trends associated with MRI-guided laser-induced thermal therapy (LITT) for intracranial tumors. Lasers Surg Med. 2013;45(6):362–9. doi: 10.1002/lsm.22151 23765325 10.1002/lsm.22151

[22] Sharma M, Habboub G, Behbahani M, Silva D, Barnett GH, Mohammadi AM. Thermal injury to corticospinal tracts and postoperative motor deficits after laser interstitial thermal therapy. Neurosurg Focus. 2016;41(4):E6 doi: 10.3171/2016.7.FOCUS16216 27690653 10.3171/2016.7.FOCUS16216

[23] Rohde GK, Aldroubi A, Dawant BM. The adaptive bases algorithm for intensity-based nonrigid image registration. IEEE Trans Med Imaging. 2003;22(11):1470–9. doi: 10.1109/TMI.2003.819299 14606680 10.1109/TMI.2003.819299

[24] Tiwari P, Danish S, Madabhushi A. Identifying MRI markers associated with early response following laser ablation for neurological disorders: preliminary findings. PLoS One. 2014;9(12):e114293 doi: 10.1371/journal.pone.0114293 25503713 10.1371/journal.pone.0114293 PMC4263602

[25] Boccardi M, Bocchetta M, Apostolova LG, Barnes J, Bartzokis G, Corbetta G, et al Delphi definition of the EADC-ADNI Harmonized Protocol for hippocampal segmentation on magnetic resonance. Alzheimers Dement. 2015;11(2):126–38. doi: 10.1016/j.jalz.2014.02.009 25130658 10.1016/j.jalz.2014.02.009 PMC4419736

[26] Frisoni GB, Jack CR Jr., Bocchetta M, Bauer C, Frederiksen KS, Liu Y, et al The EADC-ADNI Harmonized Protocol for manual hippocampal segmentation on magnetic resonance: evidence of validity. Alzheimers Dement. 2015;11(2):111–25. doi: 10.1016/j.jalz.2014.05.1756 25267715 10.1016/j.jalz.2014.05.1756 PMC4422168

[27] Zijdenbos AP, Dawant BM, Margolin RA, Palmer AC. Morphometric analysis of white matter lesions in MR images: method and validation. IEEE Trans Med Imaging. 1994;13(4):716–24. doi: 10.1109/42.363096 18218550 10.1109/42.363096

[28] Patel NV, Jethwa PR, Shetty A, Danish SF. Does the real-time thermal damage estimate allow for estimation of tumor control after MRI-guided laser-induced thermal therapy? Initial experience with recurrent intracranial ependymomas. J Neurosurg Pediatr. 2015;15(4):363–71. doi: 10.3171/2014.10.PEDS13698 25580512 10.3171/2014.10.PEDS13698

[29] Tiwari P, Danish S, Wong S, Madabhushi A. Quantitative evaluation of multi-parametric MR imaging marker changes post-laser interstitial ablation therapy (LITT) for epilepsy. Proc SPIE Int Soc Opt Eng. 2013;8671:86711Y doi: 10.1117/12.2008157 25076822 10.1117/12.2008157 PMC4112968

[30] McDannold N. Quantitative MRI-based temperature mapping based on the proton resonant frequency shift: review of validation studies. Int J Hyperthermia. 2005;21(6):533–46. doi: 10.1080/02656730500096073 16147438 10.1080/02656730500096073

[31] McNichols RJ, Gowda A, Kangasniemi M, Bankson JA, Price RE, Hazle JD. MR thermometry-based feedback control of laser interstitial thermal therapy at 980 nm. Lasers Surg Med. 2004;34(1):48–55. doi: 10.1002/lsm.10243 14755424 10.1002/lsm.10243

[32] Yeniaras E, Fuentes DT, Fahrenholtz SJ, Weinberg JS, Maier F, Hazle JD, et al Design and initial evaluation of a treatment planning software system for MRI-guided laser ablation in the brain. Int J Comput Assist Radiol Surg. 2014;9(4):659–67. doi: 10.1007/s11548-013-0948-x 24091853 10.1007/s11548-013-0948-x

[33] Jermakowicz WJ, Diaz RJ, Cass SH, Ivan ME, Komotar RJ. Use of a Mobile Intraoperative Computed Tomography Scanner for Navigation Registration During Laser Interstitial Thermal Therapy of Brain Tumors. World Neurosurg. 2016;94:418–25. doi: 10.1016/j.wneu.2016.06.126 27402436 10.1016/j.wneu.2016.06.126

