# Supplementary material for: Ablation dynamics during laser interstitial thermal therapy for mesiotemporal epilepsy
Source: PLoS One. 2018 Jul 6;13(7):e0199190. doi: 10.1371/journal.pone.0199190 (PMC6034782; doi:10.1371/journal.pone.0199190)
Supplement: S3 Table — Shown are univariate regression analyses for the 16 independent variables and τ-1 calculated from the axial TDE videos. For each variable, regression coefficient and p-value are shown. Measures with p ≤ 0.05 are bolded. AHC, amygdalohippocampal cortex; GAD, gadolinium; MTS, mesiotemporal sclerosis; M-L, mesial-lateral; S-I, superior-inferior. (DOCX) [file pone.0199190.s003.docx]

| **Variable** | ***τ^- -1^*** | | | $\boldsymbol{t}_{\boldsymbol{shift}}$ | | ***C*** | | |
| --- | --- | --- | --- | --- | --- | --- | --- | --- |
| **Age**  **Gender**  **Presence of MTS**  **CSF above**  **CSF lateral**  **AHC volume**  **T1 signal**  **T1 GAD signal**  **T2 signal**  **Ablation energy**  **Ablation time**  **Ablation power**  **M-L position**  **S-I position**  **Axial angle**  **Sagittal angle** | Coef.  0.000  - 0.001  0.000  0.000  - 0.001  0.000  0.000  - 0.001  **- 0.002**  - 0.002  - 0.001  - 0.001  0.001  - 0.001  **- 0.002**  0.001 | P  0.67  0.48  0.85  0.63  0.23  0.67  0.95  0.10  **0.01***  0.08  0.14  0.37  0.23  0.35  **0.04***  0.16 | Coef.  - 0.17  - 3.31  - 0.62  0.70  **3.30**  - 1.70  0.63  0.98  - 0.78  **33.4**  - 31.2  **- 5.46**  1.66  0.92  - 2.17  **2.89** | | P  0.84  0.14  0.81  0.56  **0.05***  0.14  0.64  0.27  0.46  **0.05***  0.06  **0.04***  0.20  0.38  0.13  **0.01*** | Coef.  - 5.79  - 13.2  - 7.53  7.34  14.5  - 11.6  5.60  6.81  12.7  12.8  11.5  11.3  - 6.39  - 10.1  6.44  - 9.48 | P  0.55  0.48  0.72  0.43  0.11  0.21  0.54  0.45  0.16  0.19  0.23  0.24  0.49  0.27  0.48  0.30 |  |
